# Diabetes-specific eating disorder and possible associated psychopathologies in adolescents with type 1 diabetes mellitus

**DOI:** 10.1007/s40519-023-01559-y

**Published:** 2023-04-08

**Authors:** Gürkan Tarçın, Hazal Akman, Didem Güneş Kaya, Nihal Serdengeçti, Sena İncetahtacı, Hande Turan, Burak Doğangün, Oya Ercan

**Affiliations:** 1 Department of Pediatric Endocrinology, Cerrahpaşa Faculty of Medicine, Istanbul University-Cerrahpaşa, Istanbul, Turkey; 2 Department of Child and Adolescent Psychiatry, Cerrahpaşa Faculty of Medicine, Istanbul University-Cerrahpaşa, Istanbul, Turkey; 3 Department of Pediatrics, Cerrahpaşa Faculty of Medicine, Istanbul University-Cerrahpaşa, Istanbul, Turkey

**Keywords:** Diabetes, Diabulimia, DEPS-R, Eating disorder, EDE-Q

## Abstract

**Purpose:**

It was aimed to investigate the frequency of the risk of diabetes-specific eating disorder (DSED) in adolescents with type 1 diabetes mellitus (T1DM) and to reveal the accompanying psychopathologies.

**Methods:**

Adolescents with T1DM aged 12–18 who applied to the pediatric diabetes outpatient clinic between July 2021 and March 2022 were included. Diabetes Eating Problem Survey-Revised (DEPS-R) was applied to all patients to determine the risk of DSED. In order to detect accompanying psychopathologies, Eating Disorder Examination Questionnaire (EDE-Q), Child Anxiety and Depression Scale-Child version (RCADS) and Parenting Style Scale were applied. After completing the scales, semi-structured interviews were conducted with all patients by a child and adolescent psychiatrist.

**Results:**

Ninety-two adolescents (45 boys, 47 girls) were included. DSED risk was found in 23.9% of the cases. A positive correlation was found between DEPS-R and EDE-Q scores (p = 0.001, rho = 0.370). RCADS mean scores were significantly higher in the group with DSED risk (p < 0.001). When the Parenting Style Scale was evaluated, psychological autonomy scores were significantly lower in the group with DSED risk (p = 0.029). As a result of the psychiatric interviews, 30 (32.6%) patients had at least 1 psychiatric disorder. Of these, 2 patients were diagnosed with eating disorder.

**Conclusion:**

Almost one-fourth of adolescents with T1DM were found to be at risk of DSED. Routine screening of adolescents with T1DM with the DEPS-R scale may provide early detection of DSED, and referral of those at risk to child psychiatry enables early diagnosis and intervention for both eating disorders and accompanying psychopathologies.

**Level of evidence:**

Level III: Evidence obtained from cohort or case-control analytic studies.

## Aim

Individuals with type 1 diabetes mellitus (T1DM) are at risk for long-term complications caused by hyperglycemia, and therefore, it is essential to maintain glycemic control throughout life [1]. Regular blood glucose measurement, appropriate dose of insulin, proper dietary regulation and portion control are needed to maintain glycemic control. Also, physical exercise is recommended, as it has beneficial effects on metabolic control [2]. Having to maintain these rules throughout a lifetime can increase the risk of developing psychopathologies, such as anxiety, depression and eating disorders [3–5]. Additionally, inadequate family support, impaired family functioning and poor communication may lead to disordered eating behaviors (DEBs) in adolescents with T1DM [6]. For early recognition of DEBs in children with T1DM, screening is recommended at the age of 10–12 years [7]. The Diabetes Eating Problems Survey-Revised (DEPS-R) is considered a useful method to screen the risk of DSED [8].

Diabulimia is a newly defined DSED that refers to the behavior of skipping insulin doses or administering insufficient doses of insulin in order to lose weight in individuals with T1DM [9]. Insulin restriction/skipping has been shown to be the most common behavior among patients with T1DM to lose weight after self-induced vomiting, excessive exercise and laxative use [10]. The weight loss in case of insulin restriction is mainly due to the loss of glucose in urine, loss of body water due to osmotic diuresis and the breakdown of fat tissue as alternative energy source [11].

This study aims to investigate the frequency of DSED risk in the adolescent age group, to determine its relationship with metabolic, anthropometric, and socio-demographic parameters, parenting styles and to reveal accompanying psychopathologies. We hypothesize that those with DSED risk would have more psychopathologies and higher anxiety and depression scores, and DSED would be associated with certain parenting styles.

## Materials and methods

This is a cross-sectional study conducted with adolescents aged 12–18 years, who have had a diagnosis of T1DM for at least 1 year and admitted to the pediatric diabetes outpatient clinic between July 2021 and March 2022.

Patients’ current age, age at diagnosis, anthropometric measurements, treatment regimens (insulin pen or pump), and HbA1c levels were obtained from their medical records. Body mass index (BMI) was calculated as the ratio of body weight to height squared (kg/m^2^) and BMI standard deviation scores (SDS) were calculated according to the standards for Turkish children [12, 13].

A questionnaire to determine the familial sociodemographic status was administered to the parents. This questionnaire included questions regarding monthly income (≤ minimum wage/between minimum wage-twice minimum wage/ ≥ twice minimum wage), the number of people living at home, and marital status (married/divorced) and educational status of parents (primary education and below/high school/university and above).

Diabetes Eating Problem Survey-Revised (DEPS-R) was applied to all patients in order to determine the risk of DSED. This questionnaire was revised for adolescents in 2010 by Markowitz et al. [8], and the adaptation, validity and reliability of the Turkish version of the questionnaire were made in 2017 [10]. The items in the questionnaire are evaluated with a six-point Likert scale (0 = never, 1 = rarely, 2 = sometimes, 3 = often, 4 = very often, 5 = always) and a score of 20 or more indicates the presence of DSED risk (DEPS-R positive). The Cronbach's alpha reliability coefficient value of the questionnaire is 0.86.

In order to detect accompanying psychopathologies, Eating Disorder Examination Questionnaire (EDE-Q), Child Anxiety and Depression Scale—Child version and Parenting Style Scale were applied to all patients.

### Parenting Style Scale (PSS)

Parenting Style Scale was used to examine the relationship between DSED and parental attitude. This scale was developed by Lamborn et al. [14] in 1991, and the validity and reliability of its Turkish version were established in 2000 [15]. The scale, consisting of 26 questions, is filled by the patients, and examines 3 attitudes: acceptance/involvement, psychological autonomy and strictness/supervision. The Cronbach Alpha internal consistency coefficients for these dimensions are 0.74, 0.75 and 0.76, respectively. The high scores obtained from the questions related to the acceptance/involvement dimension indicate that the children perceived their parents as more loving, caring, and participatory. The high score obtained from the questions related to the psychological autonomy dimension indicate a democratic parenting style and how well the child encourages himself to show his individuality. Lastly, the high scores obtained from the questions related to the strictness/supervision dimension indicate a controlling parenting style.

### Eating Disorder Examination Questionnaire (EDE-Q)

This scale was developed by Fairburn and Beglin [16] in 1944, and the validity and reliability of its Turkish version were established in 2011 [17]. It consists of subscales that reflect the severity of the eating disorder. These subscales are related to restriction, concerns about eating, body shape and weight. There are also open-ended questions about binge eating. The scale contains 28 questions and does not have a cut-off value. Higher scores indicate more severe eating disorder behaviors. The internal consistency coefficient of the scale is 0.93 and 0.70 and above for each subscale.

### The revised child anxiety and depression scale (RCADS)—child version

The RCADS was used to investigate the relationship of DSED with anxiety and depression. This scale was developed by Chorpita et al. [18] in 1944, and the validity and reliability of its Turkish version were established in 2017 [19]. It is a 47-item questionnaire developed to measure DSM-IV-based symptoms of depression and anxiety disorders in children and adolescents. RCADS has a t-score table according to age and gender in the Turkish population, which strengthens the data analysis in a wide age range. Symptoms of anxiety disorder, separation, social phobia, panic disorder, obsessive–compulsive disorder, major depressive disorder and generalized anxiety disorder are evaluated.

After completing the scales, semi-structured interviews were conducted with all patients by a child and adolescent psychiatrist. Schedule for Affective Disorders and Schizophrenia for School-Age Children-Present and Lifetime Version (K-SADS-PL), developed to determine past and present psychopathologies of children aged 6–18 years, was used in these interviews [20]. The validity and reliability of its Turkish version were established in 2018 [21].

### Statistical analyses

Statistical analyses were performed with the Statistical Package for Social Sciences (SPSS) software, version 22 (Chicago, Illinois). The variables were investigated using visual (histograms) and analytical methods (Shapiro Wilk’s test) to determine if the data were normally distributed. Student's t-test or Mann–Whitney U-test was used according to the distribution status to compare two independent groups. The Pearson chi-square test was used to examine the relation between categorical variables. The Spearman correlation test was used to evaluate the relation of DEPS-R scores with other parameters, as the DEPS-R scores were not distributed normally. The Kruskal–Wallis test was used to compare more than two independent groups. A linear regression model was developed to identify the independent predictors of DEPS-R scores. A p value of < 0.05 was considered to be statistically significant.

## Results

A total of 92 patients with T1DM (45 boys, 47 girls) were included in the study. The mean age was 15.5 ± 2.8 years, mean duration of diabetes was 6.3 ± 3.9 years, and the mean HbA1c was 8.26 ± 1.50%. 80.4% (n = 74) of the patients were receiving intensive insulin therapy using insulin pen (rapid-acting insulin with meals and long-acting insulin once a day), while 19.6% (n = 18) were using insulin pump (6 of them were advanced hybrid closed loop system). 11 patients were using a continuous glucose monitoring (CGM) device. DSED risk was found in 23.9% of the cases (n = 22, M/F: 9/13) according to the DEPS-R scale. There was no significant difference between the groups with and without DSED risk in terms of age, duration of diabetes, and HbA1c (p = 0.706, p = 0.750, p = 0.368, respectively). However, DEPS-R scores were found to be significantly higher in the subgroup with an HbA1c above 9% (n = 34) than those with less than 9% (n = 58) (p = 0.022). In addition, a positive correlation was found between HbA1c and DEPS-R scores (p = 0.016, rho = 0.253). Gender distribution was similar between the two groups (p = 0.389) (Table 1). There was no difference in DEPS-R scores between the patients using insulin pen and insulin pump (p = 0.411). A positive correlation was found between the BMI SDS of the patients and their DEPS-R scores (p = 0.003, rho = 0.304).

**Table 1:** The characteristics of the patients with and without diabetes-specific eating disorder risk

	DSED risk (+)	DSED risk (−)	*p*
Age (year)	15.7 ± 2.8	15.4 ± 2.8	0.706
Diabetes duration (year)	6.6 ± 3.3	6.3 ± 4.2	0.750
HbA1c (%)	8.52 ± 1.79	8.16 ± 1.40	0.638
Male/Female	36/34	9/13	0.389

DSED: diabetes-specific eating disorder

As for the relation with sociodemographic characteristics, the patients with divorced parents (10.9%) had significantly higher DEPS-R scores than those with married parents (p = 0.023). When the parents’ education status was examined in 3 subgroups (primary education and below/high school/university and above), no significant difference was found between these subgroups in terms of DEPS-R scores (p = 0.869 for mother’s education status and p = 0.714 for father’s education status). Lastly, no significant difference in DEPS-R scores was found between the subgroups which were created according to monthly income (p = 0.258).

A positive correlation was found between DEPS-R and EDE-Q scores (p = 0.001, rho = 0.370), and mean EDE-Q score was higher in the DEPS-R positive group than the DEPS-R negative group (p = 0.006). RCADS mean scores were significantly higher in the group with DSED risk (p < 0.001) and a positive correlation was found (p = 0.001, rho = 0.635). When the Parenting Style Scale was evaluated, psychological autonomy scores were significantly lower in the DEPS-R positive group (p = 0.029) and a negative correlation was found with DEPS-R scores (p = 0.001, rho = − 0.367). There were no differences in the acceptance/involvement and strictness/supervision dimensions between adolescents with and without DSED risk (p = 0.588 and p = 840, respectively). In the linear regression model established with the independent factors: age, RCADS score, EDE-Q score, HbA1c and gender, only RCADS score was found to be a predictor of DEPS-R scores (F = 8.724, adjusted R^2^ = 0.343, p < 0.001) (Table 2).

**Table 2:** Linear regression model to determine the predictors of Diabetes Eating Problems Survey-Revised score

	Unstandardized coefficients	Sig	95% confidence interval for B	
	B	p	Lower bound	Upper bound
Age	0.436	0.212	− 0.254	1.126
HbA1c	1.140	0.083	− 0.152	2.433
RCADS score	0.356	< 0.001	− 0.212	0.500
EDE-Q score	0.340	0.082	− 0.045	0.725
Gender	−0.765	0.682	− 4.476	2.946

RCADS: The Revised Child Anxiety and Depression Scale, EDE-Q: Eating Disorder Examination Questionnaire

**Fig. 1:**
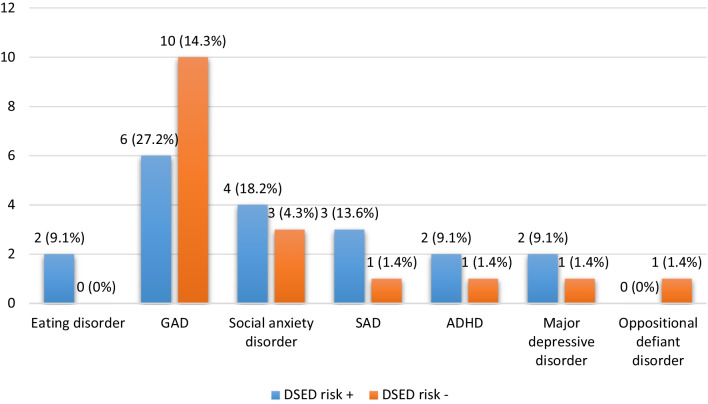
The number of the patients with psychiatric disorders according to semi-structured psychiatric interviews. DSED: diabetes-specific eating disorder; GAD: generalized anxiety disorder; SAD: separation anxiety disorder; ADHD: attention deficit hyperactivity disorder

As a result of the semi-structured psychiatric interviews, a total of 30 (32.6%) patients had at least 1 psychiatric disorder. 2 patients were diagnosed with eating disorder: One patient was diagnosed with bulimia nervosa, and the other patient with binge eating disorder. The patient with binge eating disorder was a 15-year-old girl with an HbA1c of 11.9%. She had a score of 25 in the DEPS-R scale and also skipped insulin doses most of the time. Diabulimia was considered as the definitive diagnosis in this patient. In addition, generalized anxiety disorder was diagnosed in 16, social anxiety disorder in 7, separation anxiety disorder in 4, attention deficit hyperactivity disorder in 3, major depressive disorder in 3 and oppositional defiant disorder in 1 patient. The distribution of these psychopathologies according to DSED risk is shown in Fig. 1. While 46.6% (n = 14) of patients with at least one psychiatric disorder had DEPS-R-positive, only 12.9% (n = 8) of the cases without a psychiatric disorder had it (OR [odds ratio] = 5.9, CI [confidence interval] = 2.10–16.58, p < 0.001).

## Discussion

During adolescence, physiological changes such as weight gain, increase in adipose tissue and psychosocial factors in individuals with T1DM may lead to a decrease in compliance to diabetes treatment, and thus a deterioration in metabolic control [22]. In addition, adolescents begin to explore and experiment with different lifestyles and typical adult behaviors in order to gain independence from parents and develop an individual identity. It has been suggested that experimenting with disease management is an example of exploratory behavior in this period which may result in risk-taking behaviors in diabetes management [23]. The physiological and psychological changes during adolescence can lead to dissatisfaction with body image and an increase in DEBs [10, 24]. In accordance, several studies have suggested that adolescents with T1DM have 2–3 times higher risk of eating disorders compared to their healthy peers [25–27]. In our study, the rate of adolescents at risk for DSED was found to be 23.9%. This rate has been reported between 15% and 39.3% in previous studies conducted with adolescents [28–35].

Although DSED risk has been reported to be more frequent in females, our study shows that gender is not a determinant, which is also supported by a few studies [33, 36–38]. Compared to boys, girls are more concerned with dieting and focus more on body weight, and thus, have higher risk of DEBs. Body dissatisfaction, environmental pressures regarding dietary intake, low self-esteem and desire to have cultural body ideals are the most common predisposing factors in adolescent females [5]. On the other hand, the majority of investigations in this field have mainly focused on adolescent females, and eating disorders in adolescent males often go unnoticed, despite increasing rates [39, 40]. A recent study showed a significant increase in the rate of weight reduction behaviors in European male adolescents [41].

The relation between DEBs and poor metabolic control in T1DM has previously been reported in several studies [32, 42, 43]. Skipping breakfast, binge eating, self-induced vomiting and insulin omission are related to poorer metabolic control [44]. In a meta-analysis of 11 studies, 2 of which examined the relationship of glycemic control with eating disorders and 9 with DEB, including 1363 individuals, a significant effect on both eating disorders and DEB on poor glycemic control was demonstrated [27]. Although the mean HbA1c level was found to be higher in DEPS-R positive patients in our study, it did not reach a statistically significant level. Nevertheless, there was a weak correlation between DSED risk and HbA1c levels, and it was found that the DSED risk was higher in patients with a poor metabolic control (HbA1c ≥ 9%).

In our study, it was shown that DEPS-R scores increase as the BMI increases in adolescents with T1DM, which is in line with several other studies [28–30, 33, 45]. T1DM predisposes to higher BMI, and higher BMI is associated with body dissatisfaction, which in turn results in DEBs and eating disorders [46, 47]. In adolescents with higher BMI, negative feelings about physical appearance and weight and shape concern may bring about unhealthy weight control behaviors like insulin restricting or omission. However, only a few studies have argued that BMI is not a predictor of eating disorders in adolescents with T1DM. In these studies, eating disorders were related to negative thoughts about body image and desire to lose weight [31, 38].

In the present study, the relationship between sociodemographic factors and DSED risk was investigated with a questionnaire applied to the parents. Our findings indicate that while the parents' educational status and familial economic status is not associated with DSED risk, adolescents with divorced parents are more likely to have DSED. The relation between sociodemographic characteristics and eating disorders in adolescents with T1DM has been investigated in several studies with different questionnaires [25, 33, 36, 37]. Colton et al. [25] found no relationship between family income and eating disorders in their study on adolescent girls with T1DM. In another study in which eating disorder was investigated with DEPS-R scale, as in our study, no relationship was found with the educational status of the parents [36]. Similarly, in a study conducted with DEPS (the older and longer version of DEPS-R) in adolescents with T1DM, no relationship was demonstrated with parental level of education and family structure (having one or two parents) [37]. However, another study showed that having both parents without a university degree is associated with higher DSED risk [33].

In our study, patients were also evaluated with psychiatric scales in terms of anxiety-depression and parental attitudes. In a meta-analysis, it was reported that depressive symptoms and anxiety symptoms were observed in 30.04% and 32% of adolescents with T1DM, respectively and had a negative effect on glycemic control [48]. Adolescents with T1DM have lower self-esteem than healthy peers, which brings about a higher risk for anxiety, depression and difficulty in coping with diabetes management [47]. Considering that depressive symptoms are more common in individuals with eating disorders, and eating disorders are more common in individuals with T1DM, it may be deduced that there is a relationship between these three disorders. In our study, anxiety and depression scores were higher in DEPS-R positive adolescents, as expected. This finding is in line with previous studies conducted with different anxiety and depression scales in adolescents [29, 36], also in line with an adult study [34].

Parental attitude is of great importance in the treatment adherence of adolescents with T1DM. A critical parenting style and controlling, intrusive and manipulative techniques such as love withdrawal and guilt induction have been shown to have a detrimental effect on treatment adherence, while a warm, responsive and caring parenting style has been shown to have a positive effect [49]. According to a study examining the family environment and eating disorders in adolescents with T1DM, both male and female adolescents from more cohesive families reported fewer DEBs [37]. In our study, this relation has partially been confirmed with lower psychological autonomy scores in DEPS-R positive patients though no significant relation could be determined with acceptance/involvement and strictness/supervision dimensions.

Lastly, after the completion of the scales, all patients were evaluated in terms of accompanying psychopathologies with semi-structured psychiatric interviews. A recent study reported a 5.4-fold increased risk for a positive DEPS-R score in adolescents with psychiatric disorders than those without, and the most frequent psychiatric disorders in DEPS-R positive adolescents were attention deficit and hyperactivity disorder (ADHD), anxiety disorder and eating disorders. In the current study the risk for a positive DEPS-R score is found to be 5.9-fold greater in those with psychiatric disorder than those without, and the most common accompanying psychiatric disorder was anxiety disorder, whereas eating disorder was diagnosed in only 2 of the patients.

In our study, we aimed to investigate the prevalence of DSED risk in adolescents with T1DM and to examine the accompanying psychopathologies. As a result, we found that in line with previous studies, almost one-fourth of adolescents with T1DM are at risk of DSED. Routine screening of adolescents with T1DM with the DEPS-R scale may provide early detection of DSED, and referral of those at risk to child psychiatry enables early diagnosis and intervention for both eating disorders and accompanying psychopathologies.

## Strength and limits

The strength of the study is that all cases were evaluated by a child and adolescent psychiatrist through a semi-structured interview, while in most of the previous studies, evaluations were based on psychiatric scales. Nevertheless, the eating disorder rate may have been found to be lower than the actual rate, as the patients may tend to hide some of their symptoms during a single psychiatric interview. Also, due to the small number of patients using CGM devices, data regarding CGM parameters were not given and not used in statistical analyses.

## What is already known on this subject?

Diabetes Eating Problem Survey-Revised is a useful tool to determine the risk of diabetes-specific eating disorder in patients with type 1 diabetes. Although frequency studies have been carried out many times to date, there are very few studies examining accompanying psychopathologies and the relationship of diabetes-specific eating disorder with metabolic, anthropometric and sociodemographic parameters.

## What this study adds?

Herein, diabetes-specific eating disorder risk has been found to be associated with high body mass index, poor glycemic control, anxiety and depression. Also, an increased risk has been shown in adolescents with oppressive parents who do not encourage their children to show their individuality.

## Data Availability

The datasets generated during and/or analyzed during the current study are available from the corresponding author on reasonable request.
