# Correction: Diabetes-specific eating disorder and possible associated psychopathologies in adolescents with type 1 diabetes mellitus

**DOI:** 10.1007/s40519-023-01576-x

**Published:** 2023-06-01

**Authors:** Gürkan Tarçın, Hazal Akman, Didem Güneş Kaya, Nihal Serdengeçti, Sena İncetahtacı, Hande Turan, Burak Doğangün, Oya Ercan

**Affiliations:** 1 Department of Pediatric Endocrinology, Cerrahpaşa Faculty of Medicine, Istanbul University-Cerrahpaşa, Istanbul, Turkey; 2 Department of Child and Adolescent Psychiatry, Cerrahpaşa Faculty of Medicine, Istanbul University-Cerrahpaşa, Istanbul, Turkey; 3 Department of Pediatrics, Cerrahpaşa Faculty of Medicine, Istanbul University-Cerrahpaşa, Istanbul, Turkey

**Correction: Eating and Weight Disorders - Studies on Anorexia, Bulimia and Obesity (2023) 28:36** **https://doi.org/10.1007/s40519-023-01559-y**

In this article [1], in the Methods section, we mistakenly cited Şahin et al. (reference 10) as the source for the Turkish validity and reliability of the Diabetes Eating Problem Survey-Revised, when it should have been Altinok et al. (reference 30).

In fact, we have been contacted by the author who conducted the Turkish validity and reliability study of the scale used in this study, namely Yasemin Atik Altınok, who brought this error to our attention after reading the article online. The correct reference for the Turkish validity and reliability of the scale used in this study is as follows:

Atik Altınok Y, Özgür S, Meseri R, et al. Reliability and validity of the diabetes eating problem survey in Turkish children and adolescents with type 1 diabetes mellitus. J Clin Res Pediatr Endocrinol. 2017;9(4):323–328. https://doi.org/10.4274/jcrpe.4219.

We apologize for any confusion this may have caused and confirm that this correction does not affect the results or conclusions of the study.
